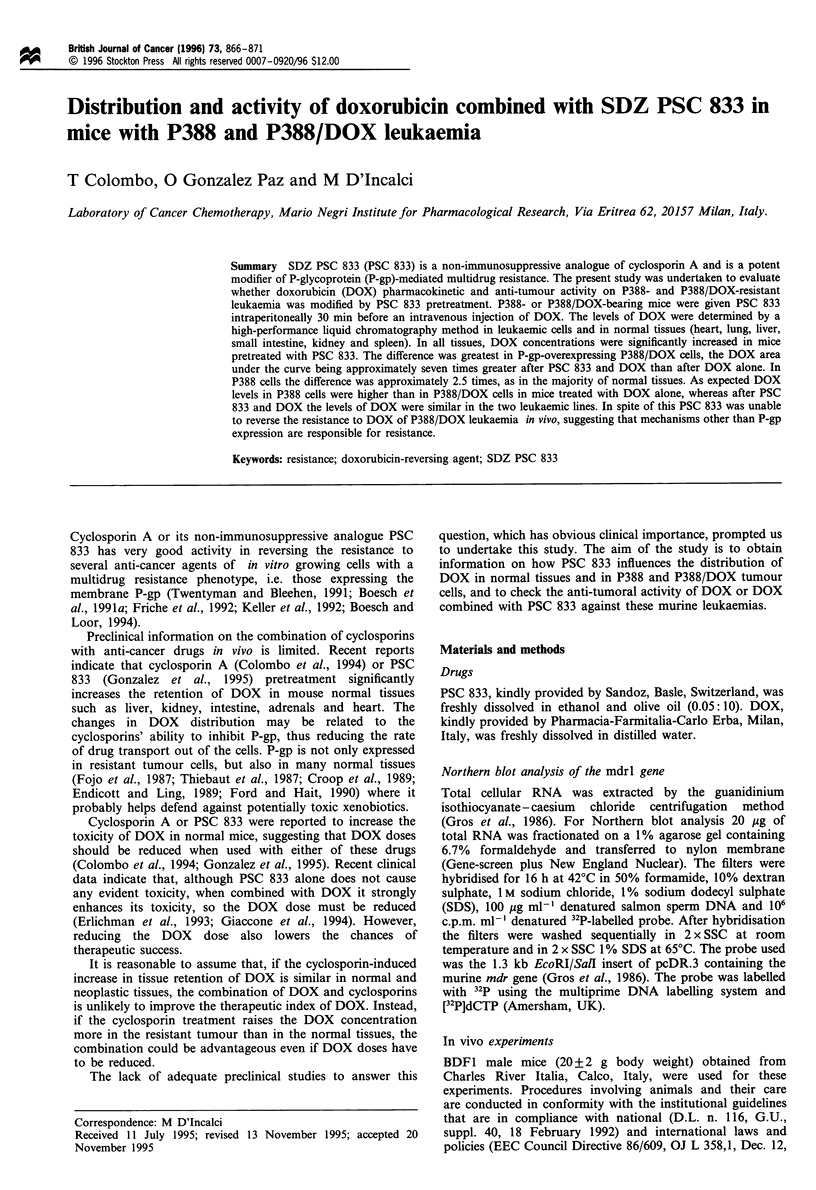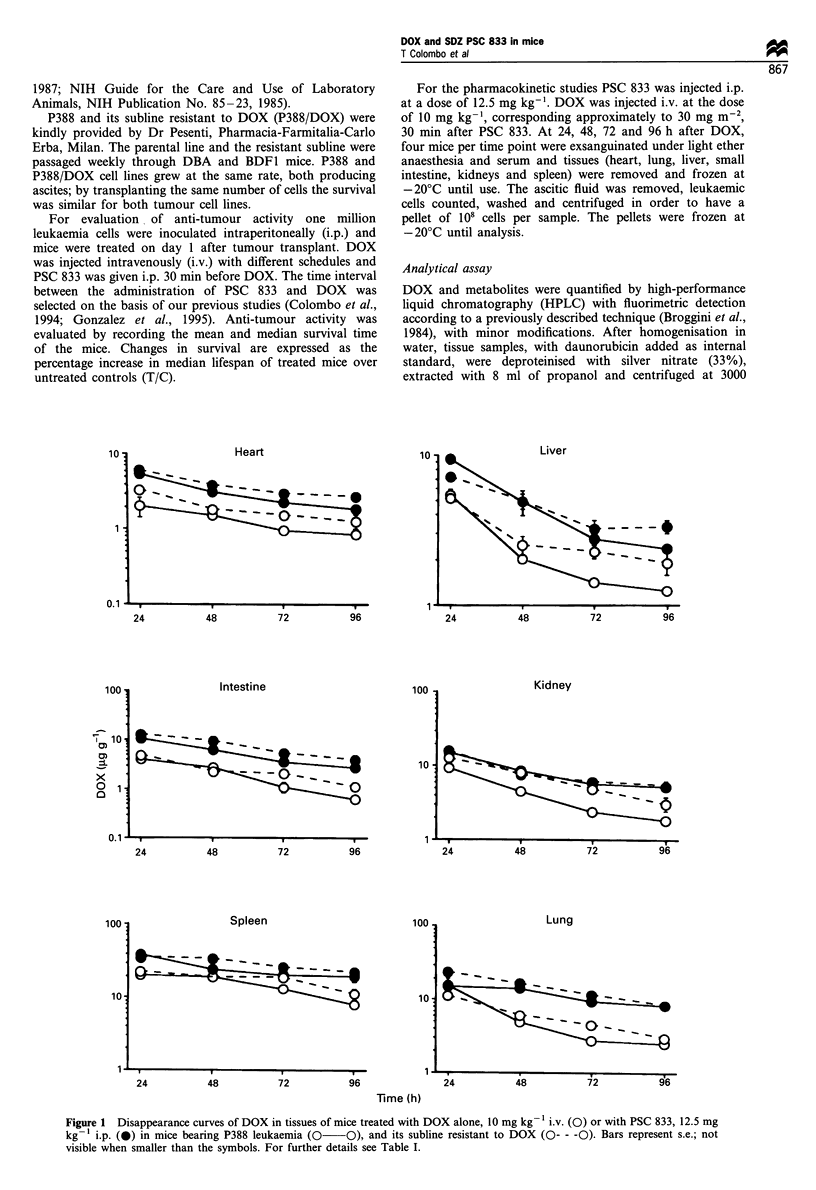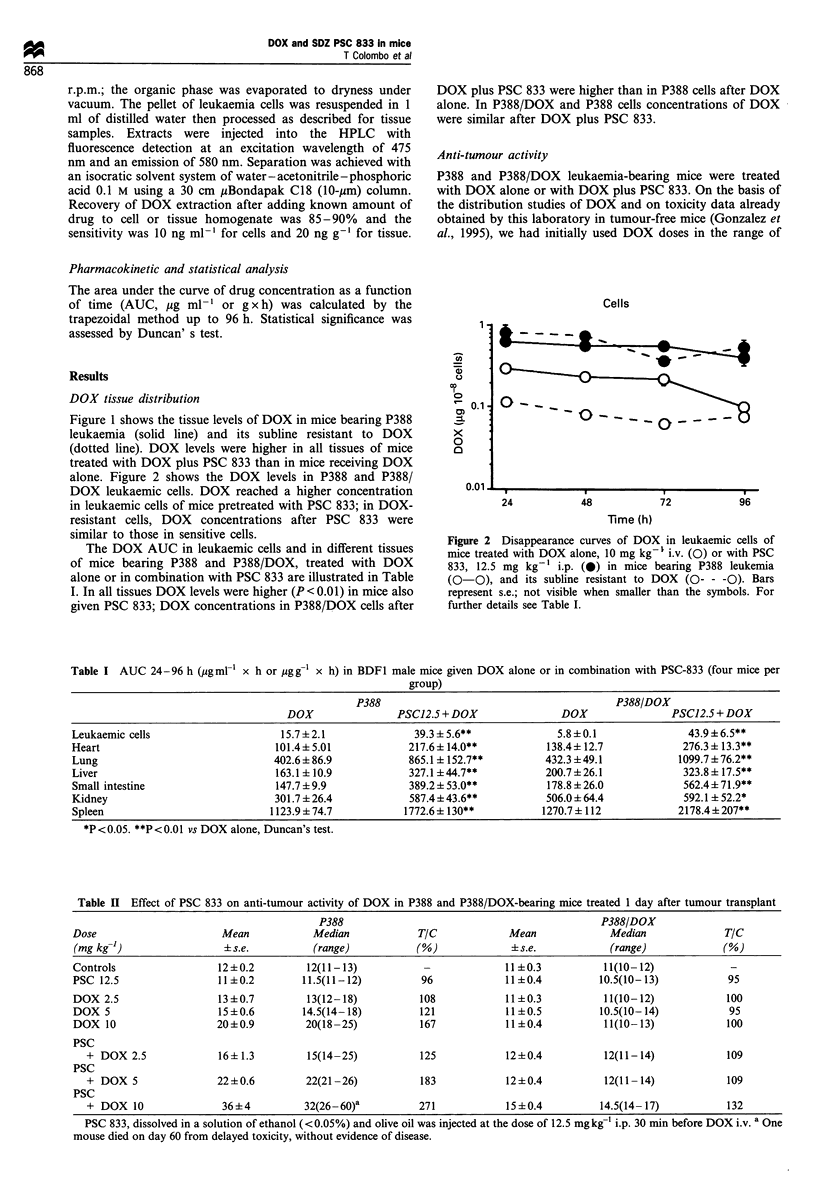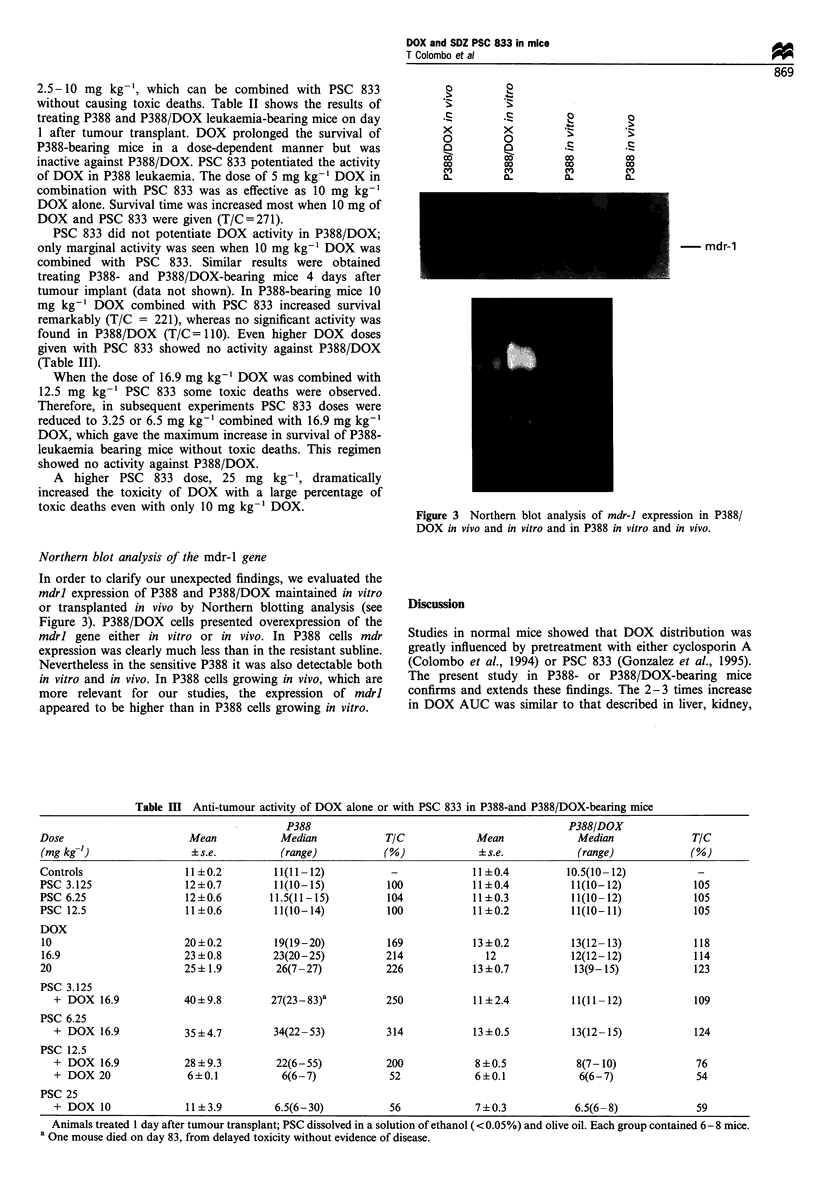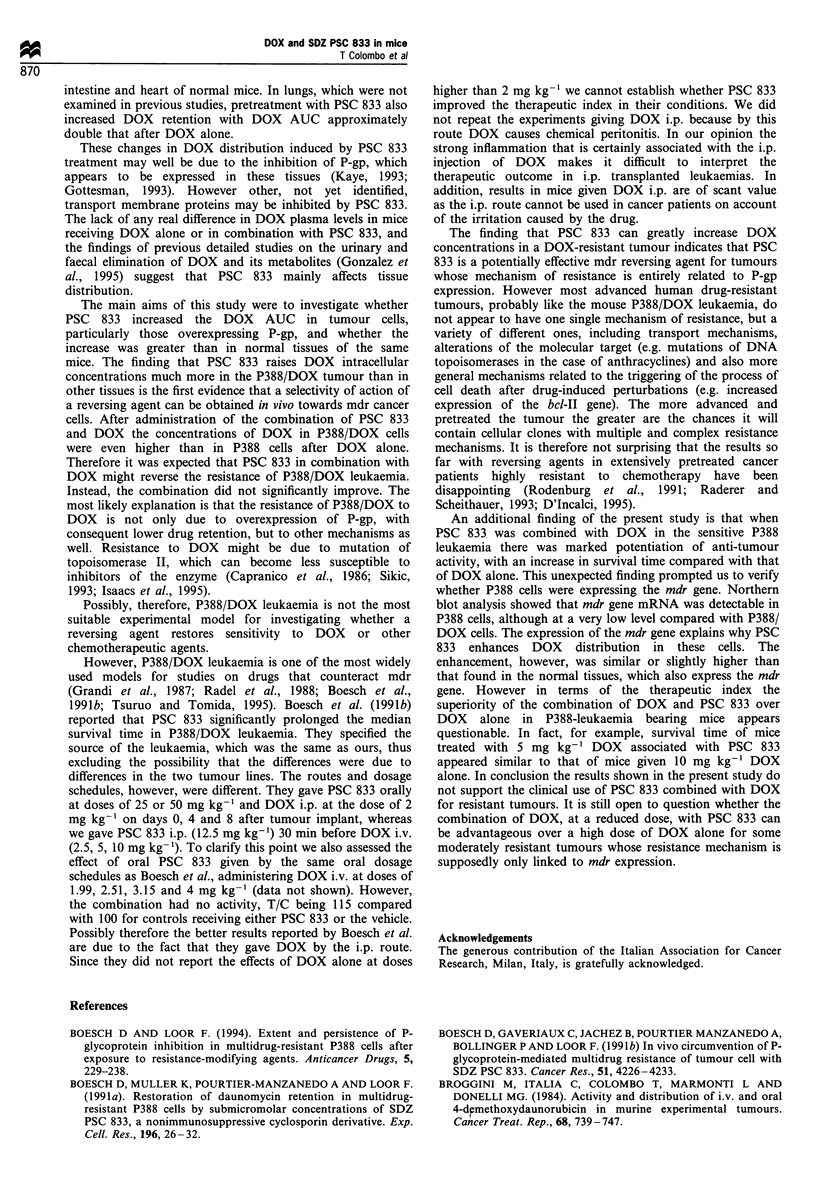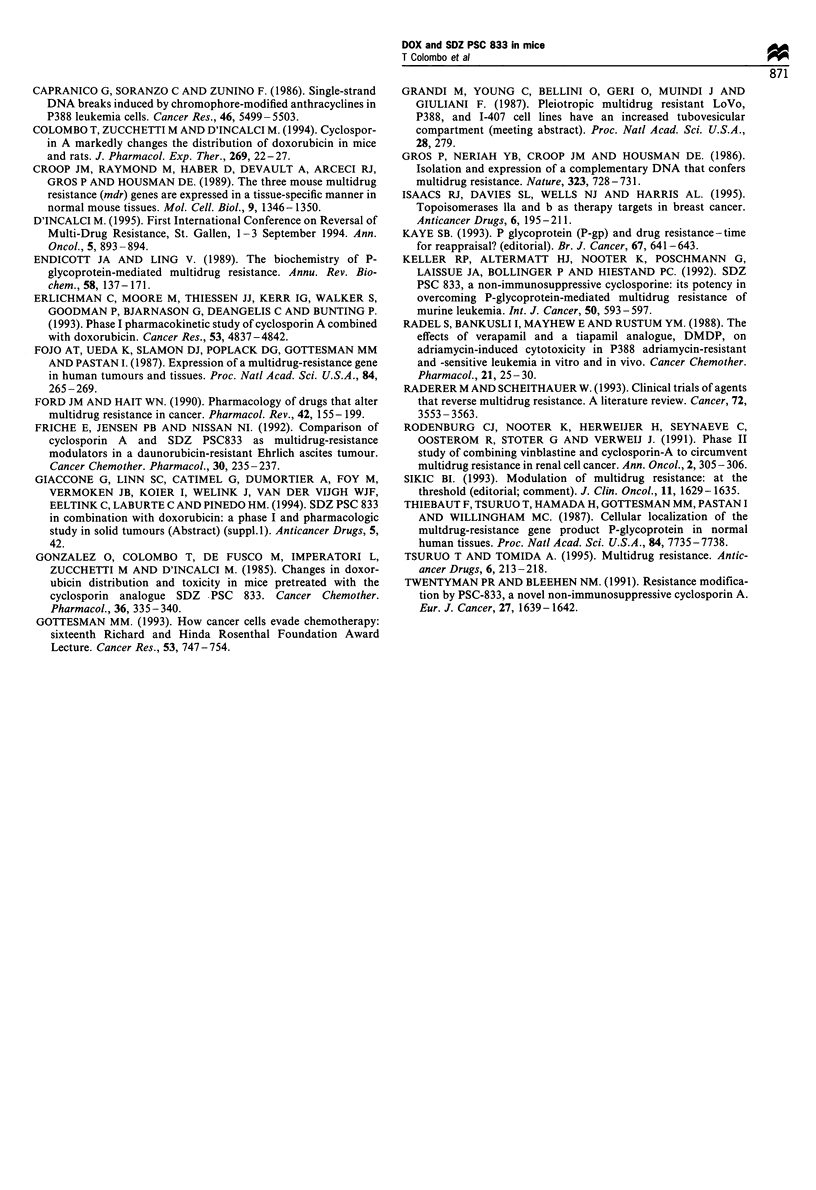# Distribution and activity of doxorubicin combined with SDZ PSC 833 in mice with P388 and P388/DOX leukaemia.

**DOI:** 10.1038/bjc.1996.154

**Published:** 1996-04

**Authors:** T. Colombo, O. Gonzalez Paz, M. D'Incalci

**Affiliations:** Laboratory of Cancer Chemotherapy, Mario Negri Institute for Pharmacological Research, Milan, Italy.

## Abstract

**Images:**


British Journal of Cancer (1996) 73, 866-871
© 1996 Stockton Press All rights reserved 0007-0920/96 $12.00

### Distribution and activity of doxorubicin combined with SDZ PSC 833 in mice with P388 and P388/DOX leukaemia

T Colombo, O Gonzalez Paz and M D'Incalci

*Laboratory of Cancer Chemotherapy, Mario Negri Institute for Pharmacological Research, Via Eritrea 62, 20157 Milan, Italy.*

**Summary** SDZ PSC 833 (PSC 833) is a non-immunosuppressive analogue of cyclosporin A and is a potent modifier of P-glycoprotein (P-gp)-mediated multidrug resistance. The present study was undertaken to evaluate whether doxorubicin (DOX) pharmacokinetic and anti-tumour activity on P388- and P388/DOX-resistant leukaemia was modified by PSC 833 pretreatment. P388- or P388/DOX-bearing mice were given PSC 833 intraperitoneally 30 min before an intravenous injection of DOX. The levels of DOX were determined by a high-performance liquid chromatography method in leukaemic cells and in normal tissues (heart, lung, liver, small intestine, kidney and spleen). In all tissues, DOX concentrations were significantly increased in mice pretreated with PSC 833. The difference was greatest in P-gp-overexpressing P388/DOX cells, the DOX area under the curve being approximately seven times greater after PSC 833 and DOX than after DOX alone. In P388 cells the difference was approximately 2.5 times, as in the majority of normal tissues. As expected DOX levels in P388 cells were higher than in P388/DOX cells in mice treated with DOX alone, whereas after PSC 833 and DOX the levels of DOX were similar in the two leukaemic lines. In spite of this PSC 833 was unable to reverse the resistance to DOX of P388/DOX leukaemia *in vivo*, suggesting that mechanisms other than P-gp expression are responsible for resistance.

**Keywords:** resistance; doxorubicin-reversing agent; SDZ PSC 833

Cyclosporin A or its non-immunosuppressive analogue PSC 833 has very good activity in reversing the resistance to several anti-cancer agents of *in vitro* growing cells with a multidrug resistance phenotype, i.e. those expressing the membrane P-gp (Twentyman and Bleehen, 1991; Boesch *et al.*, 1991*a*; Friche *et al.*, 1992; Keller *et al.*, 1992; Boesch and Loor, 1994).

Preclinical information on the combination of cyclosporins with anti-cancer drugs *in vivo* is limited. Recent reports indicate that cyclosporin A (Colombo *et al.*, 1994) or PSC 833 (Gonzalez *et al.*, 1995) pretreatment significantly increases the retention of DOX in mouse normal tissues such as liver, kidney, intestine, adrenals and heart. The changes in DOX distribution may be related to the cyclosporins' ability to inhibit P-gp, thus reducing the rate of drug transport out of the cells. P-gp is not only expressed in resistant tumour cells, but also in many normal tissues (Fojo *et al.*, 1987; Thiebaut *et al.*, 1987; Croop *et al.*, 1989; Endicott and Ling, 1989; Ford and Hait, 1990) where it probably helps defend against potentially toxic xenobiotics.

Cyclosporin A or PSC 833 were reported to increase the toxicity of DOX in normal mice, suggesting that DOX doses should be reduced when used with either of these drugs (Colombo *et al.*, 1994; Gonzalez *et al.*, 1995). Recent clinical data indicate that, although PSC 833 alone does not cause any evident toxicity, when combined with DOX it strongly enhances its toxicity, so the DOX dose must be reduced (Erlichman *et al.*, 1993; Giaccone *et al.*, 1994). However, reducing the DOX dose also lowers the chances of therapeutic success.

It is reasonable to assume that, if the cyclosporin-induced increase in tissue retention of DOX is similar in normal and neoplastic tissues, the combination of DOX and cyclosporins is unlikely to improve the therapeutic index of DOX. Instead, if the cyclosporin treatment raises the DOX concentration more in the resistant tumour than in the normal tissues, the combination could be advantageous even if DOX doses have to be reduced.

The lack of adequate preclinical studies to answer this question, which has obvious clinical importance, prompted us to undertake this study. The aim of the study is to obtain information on how PSC 833 influences the distribution of DOX in normal tissues and in P388 and P388/DOX tumour cells, and to check the anti-tumoral activity of DOX or DOX combined with PSC 833 against these murine leukaemias.

Correspondence: M D'Incalci
Received 11 July 1995; revised 13 November 1995; accepted 20 November 1995

### Materials and methods

### *Drugs*

PSC 833, kindly provided by Sandoz, Basle, Switzerland, was freshly dissolved in ethanol and olive oil (0.05:10). DOX, kindly provided by Pharmacia-Farmitalia-Carlo Erba, Milan, Italy, was freshly dissolved in distilled water.

### *Northern blot analysis of the* mdr1 *gene*

Total cellular RNA was extracted by the guanidinium isothiocyanate–caesium chloride centrifugation method (Gros *et al.*, 1986). For Northern blot analysis 20 $\mu g$ of total RNA was fractionated on a 1% agarose gel containing 6.7% formaldehyde and transferred to nylon membrane (Gene-screen plus New England Nuclear). The filters were hybridised for 16 h at 42°C in 50% formamide, 10% dextran sulphate, 1 M sodium chloride, 1% sodium dodecyl sulphate (SDS), 100 $\mu g\ ml^{-1}$ denatured salmon sperm DNA and $10^6$ c.p.m. $ml^{-1}$ denatured $^{32}P$-labelled probe. After hybridisation the filters were washed sequentially in $2\times SSC$ at room temperature and in $2\times SSC$ 1% SDS at 65°C. The probe used was the 1.3 kb *Eco*RI/*Sal*I insert of pcDR.3 containing the murine *mdr* gene (Gros *et al.*, 1986). The probe was labelled with $^{32}P$ using the multiprime DNA labelling system and [$^{32}P$]dCTP (Amersham, UK).

### In vivo *experiments*

BDF1 male mice ($20\pm2$ g body weight) obtained from Charles River Italia, Calco, Italy, were used for these experiments. Procedures involving animals and their care are conducted in conformity with the institutional guidelines that are in compliance with national (D.L. n. 116, G.U., suppl. 40, 18 February 1992) and international laws and policies (EEC Council Directive 86/609, OJ L 358,1, Dec. 12,

1987; NIH Guide for the Care and Use of Laboratory Animals, NIH Publication No. 85–23, 1985).

P388 and its subline resistant to DOX (P388/DOX) were kindly provided by Dr Pesenti, Pharmacia-Farmitalia-Carlo Erba, Milan. The parental line and the resistant subline were passaged weekly through DBA and BDF1 mice. P388 and P388/DOX cell lines grew at the same rate, both producing ascites; by transplanting the same number of cells the survival was similar for both tumour cell lines.

For evaluation of anti-tumour activity one million leukaemia cells were inoculated intraperitoneally (i.p.) and mice were treated on day 1 after tumour transplant. DOX was injected intravenously (i.v.) with different schedules and PSC 833 was given i.p. 30 min before DOX. The time interval between the administration of PSC 833 and DOX was selected on the basis of our previous studies (Colombo *et al.*, 1994; Gonzalez *et al.*, 1995). Anti-tumour activity was evaluated by recording the mean and median survival time of the mice. Changes in survival are expressed as the percentage increase in median lifespan of treated mice over untreated controls (T/C).

For the pharmacokinetic studies PSC 833 was injected i.p. at a dose of 12.5 mg $kg^{-1}$. DOX was injected i.v. at the dose of 10 mg $kg^{-1}$, corresponding approximately to 30 mg $m^{-2}$, 30 min after PSC 833. At 24, 48, 72 and 96 h after DOX, four mice per time point were exsanguinated under light ether anaesthesia and serum and tissues (heart, lung, liver, small intestine, kidneys and spleen) were removed and frozen at −20°C until use. The ascitic fluid was removed, leukaemic cells counted, washed and centrifuged in order to have a pellet of $10^8$ cells per sample. The pellets were frozen at −20°C until analysis.

*Analytical assay*

DOX and metabolites were quantified by high-performance liquid chromatography (HPLC) with fluorimetric detection according to a previously described technique (Broggini *et al.*, 1984), with minor modifications. After homogenisation in water, tissue samples, with daunorubicin added as internal standard, were deproteinised with silver nitrate (33%), extracted with 8 ml of propanol and centrifuged at 3000

**Figure 1** Disappearance curves of DOX in tissues of mice treated with DOX alone, 10 mg $kg^{-1}$ i.v. (○) or with PSC 833, 12.5 mg $kg^{-1}$ i.p. (●) in mice bearing P388 leukaemia (○——○), and its subline resistant to DOX (○- - -○). Bars represent s.e.; not visible when smaller than the symbols. For further details see Table I.

r.p.m.; the organic phase was evaporated to dryness under vacuum. The pellet of leukaemia cells was resuspended in 1 ml of distilled water then processed as described for tissue samples. Extracts were injected into the HPLC with fluorescence detection at an excitation wavelength of 475 nm and an emission of 580 nm. Separation was achieved with an isocratic solvent system of water–acetonitrile–phosphoric acid 0.1 M using a 30 cm μBondapak C18 (10-μm) column. Recovery of DOX extraction after adding known amount of drug to cell or tissue homogenate was 85–90% and the sensitivity was 10 ng $ml^{-1}$ for cells and 20 ng $g^{-1}$ for tissue.

*Pharmacokinetic and statistical analysis*

The area under the curve of drug concentration as a function of time (AUC, μg $ml^{-1}$ or g×h) was calculated by the trapezoidal method up to 96 h. Statistical significance was assessed by Duncan' s test.

### Results

*DOX tissue distribution*

Figure 1 shows the tissue levels of DOX in mice bearing P388 leukaemia (solid line) and its subline resistant to DOX (dotted line). DOX levels were higher in all tissues of mice treated with DOX plus PSC 833 than in mice receiving DOX alone. Figure 2 shows the DOX levels in P388 and P388/DOX leukaemic cells. DOX reached a higher concentration in leukaemic cells of mice pretreated with PSC 833; in DOX-resistant cells, DOX concentrations after PSC 833 were similar to those in sensitive cells.

The DOX AUC in leukaemic cells and in different tissues of mice bearing P388 and P388/DOX, treated with DOX alone or in combination with PSC 833 are illustrated in Table I. In all tissues DOX levels were higher ($P<0.01$) in mice also given PSC 833; DOX concentrations in P388/DOX cells after DOX plus PSC 833 were higher than in P388 cells after DOX alone. In P388/DOX and P388 cells concentrations of DOX were similar after DOX plus PSC 833.

*Anti-tumour activity*

P388 and P388/DOX leukaemia-bearing mice were treated with DOX alone or with DOX plus PSC 833. On the basis of the distribution studies of DOX and on toxicity data already obtained by this laboratory in tumour-free mice (Gonzalez *et al.*, 1995), we had initially used DOX doses in the range of

**Figure 2** Disappearance curves of DOX in leukaemic cells of mice treated with DOX alone, 10 mg $kg^{-1}$ i.v. (○) or with PSC 833, 12.5 mg $kg^{-1}$ i.p. (●) in mice bearing P388 leukemia (○—○), and its subline resistant to DOX (○- - -○). Bars represent s.e.; not visible when smaller than the symbols. For further details see Table I.

**Table I** AUC 24–96 h (μg$ml^{-1}$ × h or μg$g^{-1}$ × h) in BDF1 male mice given DOX alone or in combination with PSC-833 (four mice per group)

| | *P388* | | *P388/DOX* | |
|---|---|---|---|---|
| | *DOX* | *PSC12.5 + DOX* | *DOX* | *PSC12.5 + DOX* |
| Leukaemic cells | 15.7±2.1 | 39.3±5.6** | 5.8±0.1 | 43.9±6.5** |
| Heart | 101.4±5.01 | 217.6±14.0** | 138.4±12.7 | 276.3±13.3** |
| Lung | 402.6±86.9 | 865.1±152.7** | 432.3±49.1 | 1099.7±76.2** |
| Liver | 163.1±10.9 | 327.1±44.7** | 200.7±26.1 | 323.8±17.5** |
| Small intestine | 147.7±9.9 | 389.2±53.0** | 178.8±26.0 | 562.4±71.9** |
| Kidney | 301.7±26.4 | 587.4±43.6** | 506.0±64.4 | 592.1±52.2* |
| Spleen | 1123.9±74.7 | 1772.6±130** | 1270.7±112 | 2178.4±207** |

*P<0.05. **P<0.01 *vs* DOX alone, Duncan's test.

**Table II** Effect of PSC 833 on anti-tumour activity of DOX in P388 and P388/DOX-bearing mice treated 1 day after tumour transplant

| | *P388* | | | *P388/DOX* | | |
|---|---|---|---|---|---|---|
| *Dose (mg $kg^{-1}$)* | *Mean ±s.e.* | *Median (range)* | *T/C (%)* | *Mean ±s.e.* | *Median (range)* | *T/C (%)* |
| Controls | 12±0.2 | 12(11–13) | – | 11±0.3 | 11(10–12) | – |
| PSC 12.5 | 11±0.2 | 11.5(11–12) | 96 | 11±0.4 | 10.5(10–13) | 95 |
| DOX 2.5 | 13±0.7 | 13(12–18) | 108 | 11±0.3 | 11(10–12) | 100 |
| DOX 5 | 15±0.6 | 14.5(14–18) | 121 | 11±0.5 | 10.5(10–14) | 95 |
| DOX 10 | 20±0.9 | 20(18–25) | 167 | 11±0.4 | 11(10–13) | 100 |
| PSC + DOX 2.5 | 16±1.3 | 15(14–25) | 125 | 12±0.4 | 12(11–14) | 109 |
| PSC + DOX 5 | 22±0.6 | 22(21–26) | 183 | 12±0.4 | 12(11–14) | 109 |
| PSC + DOX 10 | 36±4 | 32(26–60)[a] | 271 | 15±0.4 | 14.5(14–17) | 132 |

PSC 833, dissolved in a solution of ethanol (<0.05%) and olive oil was injected at the dose of 12.5 mg$kg^{-1}$ i.p. 30 min before DOX i.v. [a] One mouse died on day 60 from delayed toxicity, without evidence of disease.

2.5–10 mg $kg^{-1}$, which can be combined with PSC 833 without causing toxic deaths. Table II shows the results of treating P388 and P388/DOX leukaemia-bearing mice on day 1 after tumour transplant. DOX prolonged the survival of P388-bearing mice in a dose-dependent manner but was inactive against P388/DOX. PSC 833 potentiated the activity of DOX in P388 leukaemia. The dose of 5 mg $kg^{-1}$ DOX in combination with PSC 833 was as effective as 10 mg $kg^{-1}$ DOX alone. Survival time was increased most when 10 mg of DOX and PSC 833 were given (T/C = 271).

PSC 833 did not potentiate DOX activity in P388/DOX; only marginal activity was seen when 10 mg $kg^{-1}$ DOX was combined with PSC 833. Similar results were obtained treating P388- and P388/DOX-bearing mice 4 days after tumour implant (data not shown). In P388-bearing mice 10 mg $kg^{-1}$ DOX combined with PSC 833 increased survival remarkably (T/C = 221), whereas no significant activity was found in P388/DOX (T/C = 110). Even higher DOX doses given with PSC 833 showed no activity against P388/DOX (Table III).

When the dose of 16.9 mg $kg^{-1}$ DOX was combined with 12.5 mg $kg^{-1}$ PSC 833 some toxic deaths were observed. Therefore, in subsequent experiments PSC 833 doses were reduced to 3.25 or 6.5 mg $kg^{-1}$ combined with 16.9 mg $kg^{-1}$ DOX, which gave the maximum increase in survival of P388-leukaemia bearing mice without toxic deaths. This regimen showed no activity against P388/DOX.

A higher PSC 833 dose, 25 mg $kg^{-1}$, dramatically increased the toxicity of DOX with a large percentage of toxic deaths even with only 10 mg $kg^{-1}$ DOX.

*Northern blot analysis of the* mdr-1 *gene*

In order to clarify our unexpected findings, we evaluated the *mdr1* expression of P388 and P388/DOX maintained *in vitro* or transplanted *in vivo* by Northern blotting analysis (see Figure 3). P388/DOX cells presented overexpression of the *mdr1* gene either *in vitro* or *in vivo*. In P388 cells *mdr* expression was clearly much less than in the resistant subline. Nevertheless in the sensitive P388 it was also detectable both *in vitro* and *in vivo*. In P388 cells growing *in vivo*, which are more relevant for our studies, the expression of *mdr1* appeared to be higher than in P388 cells growing *in vitro*.

**Figure 3** Northern blot analysis of *mdr-1* expression in P388/DOX *in vivo* and *in vitro* and in P388 *in vitro* and *in vivo*.

### Discussion

Studies in normal mice showed that DOX distribution was greatly influenced by pretreatment with either cyclosporin A (Colombo *et al*., 1994) or PSC 833 (Gonzalez *et al*., 1995). The present study in P388- or P388/DOX-bearing mice confirms and extends these findings. The 2–3 times increase in DOX AUC was similar to that described in liver, kidney,

**Table III** Anti-tumour activity of DOX alone or with PSC 833 in P388-and P388/DOX-bearing mice

| Dose ($mg\ kg^{-1}$) | P388 Mean ± s.e. | P388 Median (range) | P388 T/C (%) | P388/DOX Mean ± s.e. | P388/DOX Median (range) | P388/DOX T/C (%) |
|---|---|---|---|---|---|---|
| Controls | 11±0.2 | 11(11–12) | – | 11±0.4 | 10.5(10–12) | – |
| PSC 3.125 | 12±0.7 | 11(10–15) | 100 | 11±0.4 | 11(10–12) | 105 |
| PSC 6.25 | 12±0.6 | 11.5(11–15) | 104 | 11±0.3 | 11(10–12) | 105 |
| PSC 12.5 | 11±0.6 | 11(10–14) | 100 | 11±0.2 | 11(10–11) | 105 |
| DOX | | | | | | |
| 10 | 20±0.2 | 19(19–20) | 169 | 13±0.2 | 13(12–13) | 118 |
| 16.9 | 23±0.8 | 23(20–25) | 214 | 12 | 12(12–12) | 114 |
| 20 | 25±1.9 | 26(7–27) | 226 | 13±0.7 | 13(9–15) | 123 |
| PSC 3.125 | | | | | | |
| + DOX 16.9 | 40±9.8 | 27(23–83)[a] | 250 | 11±2.4 | 11(11–12) | 109 |
| PSC 6.25 | | | | | | |
| + DOX 16.9 | 35±4.7 | 34(22–53) | 314 | 13±0.5 | 13(12–15) | 124 |
| PSC 12.5 | | | | | | |
| + DOX 16.9 | 28±9.3 | 22(6–55) | 200 | 8±0.5 | 8(7–10) | 76 |
| + DOX 20 | 6±0.1 | 6(6–7) | 52 | 6±0.1 | 6(6–7) | 54 |
| PSC 25 | | | | | | |
| + DOX 10 | 11±3.9 | 6.5(6–30) | 56 | 7±0.3 | 6.5(6–8) | 59 |

Animals treated 1 day after tumour transplant; PSC dissolved in a solution of ethanol (<0.05%) and olive oil. Each group contained 6–8 mice.
[a] One mouse died on day 83, from delayed toxicity without evidence of disease.

intestine and heart of normal mice. In lungs, which were not examined in previous studies, pretreatment with PSC 833 also increased DOX retention with DOX AUC approximately double that after DOX alone.

These changes in DOX distribution induced by PSC 833 treatment may well be due to the inhibition of P-gp, which appears to be expressed in these tissues (Kaye, 1993; Gottesman, 1993). However other, not yet identified, transport membrane proteins may be inhibited by PSC 833. The lack of any real difference in DOX plasma levels in mice receiving DOX alone or in combination with PSC 833, and the findings of previous detailed studies on the urinary and faecal elimination of DOX and its metabolites (Gonzalez *et al.*, 1995) suggest that PSC 833 mainly affects tissue distribution.

The main aims of this study were to investigate whether PSC 833 increased the DOX AUC in tumour cells, particularly those overexpressing P-gp, and whether the increase was greater than in normal tissues of the same mice. The finding that PSC 833 raises DOX intracellular concentrations much more in the P388/DOX tumour than in other tissues is the first evidence that a selectivity of action of a reversing agent can be obtained *in vivo* towards mdr cancer cells. After administration of the combination of PSC 833 and DOX the concentrations of DOX in P388/DOX cells were even higher than in P388 cells after DOX alone. Therefore it was expected that PSC 833 in combination with DOX might reverse the resistance of P388/DOX leukaemia. Instead, the combination did not significantly improve. The most likely explanation is that the resistance of P388/DOX to DOX is not only due to overexpression of P-gp, with consequent lower drug retention, but to other mechanisms as well. Resistance to DOX might be due to mutation of topoisomerase II, which can become less susceptible to inhibitors of the enzyme (Capranico *et al.*, 1986; Sikic, 1993; Isaacs *et al.*, 1995).

Possibly, therefore, P388/DOX leukaemia is not the most suitable experimental model for investigating whether a reversing agent restores sensitivity to DOX or other chemotherapeutic agents.

However, P388/DOX leukaemia is one of the most widely used models for studies on drugs that counteract mdr (Grandi *et al.*, 1987; Radel *et al.*, 1988; Boesch *et al.*, 1991*b*; Tsuruo and Tomida, 1995). Boesch *et al.* (1991*b*) reported that PSC 833 significantly prolonged the median survival time in P388/DOX leukaemia. They specified the source of the leukaemia, which was the same as ours, thus excluding the possibility that the differences were due to differences in the two tumour lines. The routes and dosage schedules, however, were different. They gave PSC 833 orally at doses of 25 or 50 mg $kg^{-1}$ and DOX i.p. at the dose of 2 mg $kg^{-1}$ on days 0, 4 and 8 after tumour implant, whereas we gave PSC 833 i.p. (12.5 mg $kg^{-1}$) 30 min before DOX i.v. (2.5, 5, 10 mg $kg^{-1}$). To clarify this point we also assessed the effect of oral PSC 833 given by the same oral dosage schedules as Boesch *et al.*, administering DOX i.v. at doses of 1.99, 2.51, 3.15 and 4 mg $kg^{-1}$ (data not shown). However, the combination had no activity, T/C being 115 compared with 100 for controls receiving either PSC 833 or the vehicle. Possibly therefore the better results reported by Boesch *et al.* are due to the fact that they gave DOX by the i.p. route. Since they did not report the effects of DOX alone at doses higher than 2 mg $kg^{-1}$ we cannot establish whether PSC 833 improved the therapeutic index in their conditions. We did not repeat the experiments giving DOX i.p. because by this route DOX causes chemical peritonitis. In our opinion the strong inflammation that is certainly associated with the i.p. injection of DOX makes it difficult to interpret the therapeutic outcome in i.p. transplanted leukaemias. In addition, results in mice given DOX i.p. are of scant value as the i.p. route cannot be used in cancer patients on account of the irritation caused by the drug.

The finding that PSC 833 can greatly increase DOX concentrations in a DOX-resistant tumour indicates that PSC 833 is a potentially effective mdr reversing agent for tumours whose mechanism of resistance is entirely related to P-gp expression. However most advanced human drug-resistant tumours, probably like the mouse P388/DOX leukaemia, do not appear to have one single mechanism of resistance, but a variety of different ones, including transport mechanisms, alterations of the molecular target (e.g. mutations of DNA topoisomerases in the case of anthracyclines) and also more general mechanisms related to the triggering of the process of cell death after drug-induced perturbations (e.g. increased expression of the *bcl*-II gene). The more advanced and pretreated the tumour the greater are the chances it will contain cellular clones with multiple and complex resistance mechanisms. It is therefore not surprising that the results so far with reversing agents in extensively pretreated cancer patients highly resistant to chemotherapy have been disappointing (Rodenburg *et al.*, 1991; Raderer and Scheithauer, 1993; D'Incalci, 1995).

An additional finding of the present study is that when PSC 833 was combined with DOX in the sensitive P388 leukaemia there was marked potentiation of anti-tumour activity, with an increase in survival time compared with that of DOX alone. This unexpected finding prompted us to verify whether P388 cells were expressing the *mdr* gene. Northern blot analysis showed that *mdr* gene mRNA was detectable in P388 cells, although at a very low level compared with P388/DOX cells. The expression of the *mdr* gene explains why PSC 833 enhances DOX distribution in these cells. The enhancement, however, was similar or slightly higher than that found in the normal tissues, which also express the *mdr* gene. However in terms of the therapeutic index the superiority of the combination of DOX and PSC 833 over DOX alone in P388-leukaemia bearing mice appears questionable. In fact, for example, survival time of mice treated with 5 mg $kg^{-1}$ DOX associated with PSC 833 appeared similar to that of mice given 10 mg $kg^{-1}$ DOX alone. In conclusion the results shown in the present study do not support the clinical use of PSC 833 combined with DOX for resistant tumours. It is still open to question whether the combination of DOX, at a reduced dose, with PSC 833 can be advantageous over a high dose of DOX alone for some moderately resistant tumours whose resistance mechanism is supposedly only linked to *mdr* expression.

**Acknowledgements**
The generous contribution of the Italian Association for Cancer Research, Milan, Italy, is gratefully acknowledged.

### References

BOESCH D AND LOOR F. (1994). Extent and persistence of P-glycoprotein inhibition in multidrug-resistant P388 cells after exposure to resistance-modifying agents. *Anticancer Drugs*, **5**, 229–238.

BOESCH D, MULLER K, POURTIER-MANZANEDO A AND LOOR F. (1991*a*). Restoration of daunomycin retention in multidrug-resistant P388 cells by submicromolar concentrations of SDZ PSC 833, a nonimmunosuppressive cyclosporin derivative. *Exp. Cell. Res.*, **196**, 26–32.

BOESCH D, GAVERIAUX C, JACHEZ B, POURTIER MANZANEDO A, BOLLINGER P AND LOOR F. (1991*b*) In vivo circumvention of P-glycoprotein-mediated multidrug resistance of tumour cell with SDZ PSC 833. *Cancer Res.*, **51**, 4226–4233.

BROGGINI M, ITALIA C, COLOMBO T, MARMONTI L AND DONELLI MG. (1984). Activity and distribution of i.v. and oral 4-demethoxydaunorubicin in murine experimental tumours. *Cancer Treat. Rep.*, **68**, 739–747.

CAPRANICO G, SORANZO C AND ZUNINO F. (1986). Single-strand DNA breaks induced by chromophore-modified anthracyclines in P388 leukemia cells. *Cancer Res.*, **46**, 5499–5503.

COLOMBO T, ZUCCHETTI M AND D'INCALCI M. (1994). Cyclosporin A markedly changes the distribution of doxorubicin in mice and rats. *J. Pharmacol. Exp. Ther.*, **269**, 22–27.

CROOP JM, RAYMOND M, HABER D, DEVAULT A, ARCECI RJ, GROS P AND HOUSMAN DE. (1989). The three mouse multidrug resistance (*mdr*) genes are expressed in a tissue-specific manner in normal mouse tissues. *Mol. Cell. Biol.*, **9**, 1346–1350.

D'INCALCI M. (1995). First International Conference on Reversal of Multi-Drug Resistance, St. Gallen, 1–3 September 1994. *Ann. Oncol.*, **5**, 893–894.

ENDICOTT JA AND LING V. (1989). The biochemistry of P-glycoprotein-mediated multidrug resistance. *Annu. Rev. Biochem.*, **58**, 137–171.

ERLICHMAN C, MOORE M, THIESSEN JJ, KERR IG, WALKER S, GOODMAN P, BJARNASON G, DEANGELIS C AND BUNTING P. (1993). Phase I pharmacokinetic study of cyclosporin A combined with doxorubicin. *Cancer Res.*, **53**, 4837–4842.

FOJO AT, UEDA K, SLAMON DJ, POPLACK DG, GOTTESMAN MM AND PASTAN I. (1987). Expression of a multidrug-resistance gene in human tumours and tissues. *Proc. Natl Acad. Sci. U.S.A.*, **84**, 265–269.

FORD JM AND HAIT WN. (1990). Pharmacology of drugs that alter multidrug resistance in cancer. *Pharmacol. Rev.*, **42**, 155–199.

FRICHE E, JENSEN PB AND NISSAN NI. (1992). Comparison of cyclosporin A and SDZ PSC833 as multidrug-resistance modulators in a daunorubicin-resistant Ehrlich ascites tumour. *Cancer Chemother. Pharmacol.*, **30**, 235–237.

GIACCONE G, LINN SC, CATIMEL G, DUMORTIER A, FOY M, VERMOKEN JB, KOIER I, WELINK J, VAN DER VIJGH WJF, EELTINK C, LABURTE C AND PINEDO HM. (1994). SDZ PSC 833 in combination with doxorubicin: a phase I and pharmacologic study in solid tumours (Abstract) (suppl.1). *Anticancer Drugs*, **5**, 42.

GONZALEZ O, COLOMBO T, DE FUSCO M, IMPERATORI L, ZUCCHETTI M AND D'INCALCI M. (1985). Changes in doxorubicin distribution and toxicity in mice pretreated with the cyclosporin analogue SDZ PSC 833. *Cancer Chemother. Pharmacol.*, **36**, 335–340.

GOTTESMAN MM. (1993). How cancer cells evade chemotherapy: sixteenth Richard and Hinda Rosenthal Foundation Award Lecture. *Cancer Res.*, **53**, 747–754.

GRANDI M, YOUNG C, BELLINI O, GERI O, MUINDI J AND GIULIANI F. (1987). Pleiotropic multidrug resistant LoVo, P388, and I-407 cell lines have an increased tubovesicular compartment (meeting abstract). *Proc. Natl Acad. Sci. U.S.A.*, **28**, 279.

GROS P, NERIAH YB, CROOP JM AND HOUSMAN DE. (1986). Isolation and expression of a complementary DNA that confers multidrug resistance. *Nature*, **323**, 728–731.

ISAACS RJ, DAVIES SL, WELLS NJ AND HARRIS AL. (1995). Topoisomerases lla and b as therapy targets in breast cancer. *Anticancer Drugs*, **6**, 195–211.

KAYE SB. (1993). P glycoprotein (P-gp) and drug resistance–time for reappraisal? (editorial). *Br. J. Cancer*, **67**, 641–643.

KELLER RP, ALTERMATT HJ, NOOTER K, POSCHMANN G, LAISSUE JA, BOLLINGER P AND HIESTAND PC. (1992). SDZ PSC 833, a non-immunosuppressive cyclosporine: its potency in overcoming P-glycoprotein-mediated multidrug resistance of murine leukemia. *Int. J. Cancer*, **50**, 593–597.

RADEL S, BANKUSLI I, MAYHEW E AND RUSTUM YM. (1988). The effects of verapamil and a tiapamil analogue, DMDP, on adriamycin-induced cytotoxicity in P388 adriamycin-resistant and -sensitive leukemia in vitro and in vivo. *Cancer Chemother. Pharmacol.*, **21**, 25–30.

RADERER M AND SCHEITHAUER W. (1993). Clinical trials of agents that reverse multidrug resistance. A literature review. *Cancer*, **72**, 3553–3563.

RODENBURG CJ, NOOTER K, HERWEIJER H, SEYNAEVE C, OOSTEROM R, STOTER G AND VERWEIJ J. (1991). Phase II study of combining vinblastine and cyclosporin-A to circumvent multidrug resistance in renal cell cancer. *Ann. Oncol.*, **2**, 305–306.

SIKIC BI. (1993). Modulation of multidrug resistance: at the threshold (editorial; comment). *J. Clin. Oncol.*, **11**, 1629–1635.

THIEBAUT F, TSURUO T, HAMADA H, GOTTESMAN MM, PASTAN I AND WILLINGHAM MC. (1987). Cellular localization of the multdrug-resistance gene product P-glycoprotein in normal human tissues. *Proc. Natl Acad. Sci. U.S.A.*, **84**, 7735–7738.

TSURUO T AND TOMIDA A. (1995). Multidrug resistance. *Anticancer Drugs*, **6**, 213–218.

TWENTYMAN PR AND BLEEHEN NM. (1991). Resistance modification by PSC-833, a novel non-immunosuppressive cyclosporin A. *Eur. J. Cancer*, **27**, 1639–1642.